# Antibiotics in malaria therapy: which antibiotics except tetracyclines and macrolides may be used against malaria?

**DOI:** 10.1186/s12936-016-1613-y

**Published:** 2016-11-15

**Authors:** Tiphaine Gaillard, Marylin Madamet, Francis Foguim Tsombeng, Jérôme Dormoi, Bruno Pradines

**Affiliations:** 1 Fédération des Laboratoires, Hôpital d’Instruction des Armées Saint Anne, Toulon, France; 2 Unité de Parasitologie et d’Entomologie, Département des Maladies Infectieuses, Institut de Recherche Biomédicale des Armées, Marseille, France; 3 Unité de Recherche sur les Maladies Infectieuses et Tropicales Emergentes, Aix Marseille Université, UM 63, CNRS 7278, IRD 198, Inserm 1095, Marseille, France; 4 Centre National de Référence du Paludisme, Marseille, France

**Keywords:** Antibiotics, Malaria, *Plasmodium falciparum*, Anti-malarial drug, Resistance, Prophylaxis, Treatment

## Abstract

Malaria, a parasite vector-borne disease, is one of the most significant health threats in tropical regions, despite the availability of individual chemoprophylaxis. Malaria chemoprophylaxis and chemotherapy remain a major area of research, and new drug molecules are constantly being developed before drug-resistant parasites strains emerge. The use of anti-malarial drugs is challenged by contra-indications, the level of resistance of *Plasmodium falciparum* in endemic areas, clinical tolerance and financial cost. New therapeutic approaches are currently needed to fight against this disease. Some antibiotics that have shown potential effects on malaria parasite have been recently studied in vitro or in vivo intensively. Two families, tetracyclines and macrolides and their derivatives have been particularly studied in recent years. However, other less well-known have been tested or are being used for malaria treatment. Some of these belong to older families, such as quinolones, co-trimoxazole or fusidic acid, while others are new drug molecules such as tigecycline. These emerging antibiotics could be used to prevent malaria in the future. In this review, the authors overview the use of antibiotics for malaria treatment.

## Background

Malaria in one of the most significant health threats in tropical regions with high morbidity and mortality rates. In 2015, approximately 3.2 billion people—nearly half of the world’s population—were at risk of malaria [1]. According to the latest WHO estimates, released in December 2015, there were 214 million cases of malaria in 2015 and 438,000 deaths. Sub-Saharan Africa continues to carry a disproportionately high share of the global malaria burden. In 2015, the region was home to 88% of malaria cases and 90% of malaria deaths. Despite the availability of chemotherapy and chemoprophylaxis, the disease remains an important public health problem in many countries. The spread of anti-malarial drug resistance from Southeast Asia to Africa has previously happened with chloroquine and sulfadoxine-pyrimethamine [2, 3]. To overcome this problem new therapeutic strategies are been developed. Many other drugs formulations have recently been developed such as combination of molecules (artemisinin-based combination therapy) [4] and use of antibiotics that have been shown to be effective against malaria parasites [5, 6].

This literature review focused on the use of antibiotics for malaria chemoprophylaxis and treatment. The term of antibiotic in this review defined any compound that has been used to treat bacterial infections and their analogues developed if there were active against *Plasmodium falciparum*. Two families, tetracyclines and macrolides and their derivatives, have been the focus of many studies in the past 30 years and were previously described in two reviews [5, 6]. However, other antibiotics against malaria parasites could be developed in the future. This third and final review on the use of antibiotics as anti-malarial drugs described the activity against *P. falciparum* of co-trimoxazole, quinolones, tigecycline, mirincamycin, ketolides, fusidic acid and thiopeptides.

## Co-trimoxazole

Co-trimoxazole is a combination of trimethoprim and sulfamethoxazole. Trimethoprim is derived from pyrimidine and belongs to a group of compounds well characterized for their antibacterial activity. Trimethoprim inhibits the enzyme dihydrofolate reductase, and has been shown to act as a sulfonamide potentiator [7]. In 1971, a combination of trimethoprim and sulfamethoxazole was reported to be effective in the treatment of malaria infections in semi-immune Nigerian children [8]. Additionally, co-trimoxazole prophylaxis is currently recommended by the World Health Organization (WHO) to prevent opportunistic infections in HIV infected persons [9]. Considering the reports on the impact of co-trimoxazole on malaria, both in HIV-infected and healthy individuals, the efficacy of this drug was studied for a potential use in both prophylaxis and treatment of malaria [10, 11]. It appears that co-trimoxazole could be an alternative for malaria treatment. Additionally, co-trimoxazole could also be a good alternative in malaria prophylaxis for different target groups, including children and adults, HIV positive or negative patients and pregnant women [12–15]. Daily use of co-trimoxazole during pregnancy had similar effects as intermittent preventive treatment (IPT) in terms of preterm deliveries, stillbirths, neonatal deaths, spontaneous abortions or birth weights [14]. Co-trimoxazole is effective (above 90%) for uncomplicated malaria treatment in children in areas of high endemicity [12, 16–18]. However, co-trimoxazole had no gametocytocidal effect in vivo [8, 15] and neither trimethroprim nor sulfamethoxazole have action in vitro against early and late stage gametocytes [19]. Additionally, the use of this combination in non-pregnant HIV-infected patients reduced the risk of malaria incidence in children and adults in Africa [20–25]. In Uganda, despite the high rates of antimicrobial resistance to co-trimoxazole, its use in non-pregnant HIV-infected patients reduced the risk of mortality by 46–63% and was associated to lower rates of malaria infection [23, 24]. In a study assessing the prophylaxis effect of co-trimoxazole, its use in combination with insecticide-treated bed nets reduced the risk of malaria incidence by at least 95% [13, 22]. The same result was observed in Mali within non-pregnant HIV-negative population with a protective efficacy of 99.5 and 97% in symptomatic and asymptomatic populations, respectively [26]. In an area with high malaria transmission rate and high antifolate resistance such as Uganda, daily prophylaxis with co-trimoxazole yielded a 39% reduction of malaria incidence in children [27]. During pregnancy, malaria prophylaxis with co-trimoxazole reduced malaria incidence in HIV-pregnant women as well as the prevalence of placental malaria [28, 29]. Similar results were observed among HIV-infected pregnant women on neonatal mortality in Zambia (from 9 to 0%) [30].

Concerning the resistance to co-trimoxazole, some studies have shown evidence of cross-resistance between co-trimoxazole and sulfadoxine-pyrimethamine [31, 32]. However, co-trimoxazole remains effective in areas with high antifolate resistance, and it seems not to be associated with a higher prevalence of mutations related to antifolate resistance [21, 27, 33]. The use of co-trimoxazole as prophylaxis in HIV-infected patients did not show any selection of resistant *P. falciparum* parasites to sulfadoxine-pyrimethamine among HIV-uninfected household members [34]. Data from another study conducted in Kenya have shown that the daily use of co-trimoxazole prevented malaria and reduced the prevalence of resistant *P. falciparum* parasites to sulfadoxine-pyrimethamine despite the fact that it increased pneumococcus and *Escherichia coli* resistance [35].

Additionally, co-trimoxazole is inexpensive, almost universally available and has a wide clinical spectrum of activity against bacteria, fungi and protozoan infections [36]. Co-trimoxazole could be an alternative for IPT, but not associated with sulfadoxine-pyrimethamine. Indeed, in HIV-infected women, IPT by sulfadoxine-pyrimethamine is contraindicated to avoid the potentially serious drug interactions with co-trimoxazole, which is currently recommended in all HIV-infected pregnant women to prevent opportunistic infections. The paradox is that HIV-infected women who are the most vulnerable to malaria infection cannot be treated with sulfadoxine-pyrimethamine as IPT, even if the use of sulfadoxine-pyrimethamine is a life-saving and highly cost-effective intervention [37]. However, most information available on co-trimoxazole comes from Africa; more randomized controlled trials, including trials from other areas, are needed to evaluate the efficacy and the safety of co-trimoxazole.

## Anti-malarial quinolones

Quinolones are synthetic compounds mostly used as antibiotics for their bactericidal properties. Quinolones contain the 4-oxo-1,4-dihydroquinoline skeleton. The first antibacterial quinolone, nalidixic acid, was discovered as a by-product during the synthesis of chloroquine [38]. The quinolone scaffold is present in the structure of compounds that display anti-malarial activity, probably by targeting the gyrase enzyme of the parasites, a type II topo-isomerase [39]. However, in *P. falciparum* there is evidence for off-target toxicity, particularly for ciprofloxacin [40]. The main quinolones used as antibiotics are fluoroquinolones. Their anti-malarial activity has been documented [41]. Norfloxacin, ofloxacin, pefloxacin and trovafloxacin displayed low in vitro anti-malarial activity in the micromolar range against *P. falciparum* (20– >100 µM) [42–46]. Many studies have shown that ciprofloxacin displayed the best in vitro anti-malarial activity within the first 48 h [47–49] Prolonged exposure of parasites to ciprofloxacin increased its anti-malarial activity [47–50]. Additionally, this antibiotic has an in vitro potentiating effect on primaquine by increasing its anti-malarial activity [51]. Chemical modifications of ciprofloxacin by combination of organometallic compounds and a pro-drug approach have been performed to improve its malarial activity [52]. Recently, the graft of a ferrocenic moiety to ciprofloxacin led to an additional enhancement of activity, which could be attributed, first, to oxidative stress due to the redox properties of the ferrocene/ferricenium and, second, to the high lipophilicity of the ferrocene, which may help transport drugs across membranes [53].

With 160 mg/kg, ciprofloxacin was able to prevent mortality of 85% of mice infected with *Plasmodium yoelii* [54]. However, the high doses used and the frequent administrations (every 8 h for 3 days) to achieve effect showed the poor efficacy of ciprofloxacin used alone. Ciprofloxacin was shown to also potentiate the in vivo efficacy of amodiaquine, mefloquine and artemisinin derivatives and chloroquine in mice infected with *Plasmodium berghei* [55–58].

However, there are some evidences from different clinical trials studies that fluoroquinolones do not have promising results for uncomplicated malaria treatment. In a study conducted in Thailand, oral ciprofloxacin at 750 mg every 12 h for seven days failed to cure uncomplicated falciparum malaria [59]. Additionally, norfloxacin at 400 mg twice a day for three days cured only 40% of the patients, while 100% of the patients treated with chloroquine were cured [60]. It is clear that fluoroquinolones used alone are not a good antimalarial drug and should always be associated with other standard drugs.

The subsequent discovery of an apicoplast in *Plasmodium* increased interest in further elucidating the mode of action of fluoroquinolones on this parasite [61]. In this context, it has been shown that ciprofloxacin affects *P. falciparum* by causing the formation of an abnormal apicoplast and a ‘delayed death’ of treated parasites [62]. Quinolones display anti-malarial activity, probably by targeting the gyrase of *P. falciparum,* an enzyme that is involved in apicoplast DNA replication [39, 40]. However, *in P. falciparum* there is evidence for off-target toxicity, particularly for ciprofloxacin [40]. Ciprofloxacin also reduced the expression of the *P. falciparum* single-stranded DNA binding protein (PfSSB protein), which plays an essential role in many aspects of nucleic acid metabolism, including DNA replication, recombination and repair [63]. Despite the low activity of fluoroquinolones on malaria parasites, new fluoroquinolone analogues molecules displayed better in vitro activity against *P. falciparum* than ciprofloxacin and synergized the activity of artemisinin [64, 65].

Recent research has demonstrated the promising potential of quinolone analogues as anti-malarial drugs. They show very good efficacy and target several stages of the malaria parasite life cycle, including the erythrocytic, hepatic and gametocytes stages.

Additionally, they present new modes of action that differ from those of most currently used drugs. Compounds with quinolone scaffolds are classified into different structural families: endochin and its analogues, acridinones, haloalkoxyacridinones, carboxyquinolones, 4(1H)-quinolones, and quinolone-3-diarylethers.

Anti-malarial property of endochin, synthesized from the nucleus of the quinolones, was first demonstrated on avian malaria in the late 1940s [66]. Further studies on this molecule have established its activity against both hepatic and erythrocytic stages of malaria parasites. However, endochin has proven to be ineffective in vivo against human malaria due to its inactivation by cytochrome P450 enzymes [67]. Endochin analogues targeted the *Plasmodium* bc_1_ complex at a low nanomolar range, as atovaquone does [68]. New endochin analogues with improved anti-malarial properties were synthesized. The insertion of two fluorine atoms (ELQ-121) or one chlorine atom (ELQ-130) in the endochin led to metabolic stability in the presence of cytochrome P450 compared to the parent endochin [67]. The polyethylene glycol of ELQ-121 (ELQ-125) displayed higher oral bio-availability than both endochin and ELQ-121 and cured infected mice with *P. yoelii* at a dose of 50 mg/kg/day in three days, whereas endochin showed no efficacy [67]. Recently, new substituted compounds based on endochin with a 4(1*H*)-quinolone scaffold displayed activity against multi-drug resistant *P. falciparum* isolates at low nanomolar range. They targeted the *Plasmodium* bc_1_ complex without cross-resistance with atovaquone, demonstrated erythrocytic and exoerythrocytic activity and transmission blocking, and improved metabolic stability in the presence of cytochrome P450 [69–74]. Some of these analogues (quinolone-3-diarylethers) were also highly active against *Plasmodium vivax* isolates [75]. Additionally, hydroxyl-2-dodecyl-4-(1*H*) quinolone displayed a dual mechanism of action against two respiratory enzymes, the *P. falciparum* bc_1_ complex and the *P. falciparum* type II NADH: ubiquinone oxydoreductase (PfNDH2) [76]. Studies were undertaken to identify lead compounds with high activity against PfNDH2. Several analogues of quinolones showed the capacity to inhibit PfNDH2 at a low nanomolar range [77, 78].

Tricyclic acridinones are structurally related to quinolones with the 4-oxo-1,4-dihydroquinolone skeleton. Their anti-malarial activity was first reported in 1947 [79]. Other molecules with improved therapeutic properties were later discovered. Because of their poor aqueous solubility and metabolic instability, acridinones were not further evaluated [80]. Recent studies aimed at re-evaluating the acridinones which seem promising, with IC_50_ <20 nM [81]. Acridinones, like quinolones, targeted the cytochrome bc_1_ complex and also inhibited haem polymerization, as chloroquine does [82].

Additionally, acridinones blocked the transmission to mosquitoes by preventing oocysts development [72].

The haloalkoxyacridinones are a new class of acridinone [83]. Some of them exhibit extraordinarily strong anti-malarial activity in vitro, with favourable IC_50_ values of 1 μM [84].

Since the discovery of the anti-malarial activity of ICI56-780, a carboxyl derivative of quinolones [85], screening of new compounds has led to the discovery of various 3-carboxyquinolones, which displayed in vitro activity <100 nM against *P. falciparum* strains and targeted the cytochrome bc_1_ complex and the dihydroorotate dehydrogenase (DHODH) [86, 87].

More research is required to optimize the pharmacodynamic properties of all these derivatives of quinolones. A significant obstacle to the clinical development of these new quinolone analogues is related to their physicochemical properties. Their relatively poor aqueous solubility limits absorption. This implies that only low blood concentrations can be achieved following oral dosing. Pro-drug strategy represents a viable approach to improve bio-availability and overcome the physicochemical limitations of quinolone analogues to deliver the active drug at concentrations sufficient for safety, as well as achieving single-dose cures [88]. At the molar equivalent dose of 3 mg/kg of body weight, the delivery of ELQ-300, a endochin analogue from ELQ-337, its O-linked carbonate ester pro-drug, is enhanced by threefold to fourfold, reaching a maximum concentration of drug in serum [88].

The very good efficacy at nanomolar range against *P. falciparum* and *P. vivax*, their action on several stages of the malaria parasite life cycle, including the erythrocytic, hepatic and gametocytes stages, the new modes of action that differ from those of most currently used drugs demonstrated the promising potential of these new quinolone analogues as anti-malarial drugs to treat malaria. Further studies should be promoted to assess the effects of these new quinolones in combination with artemisinin derivatives and other anti-malarial drugs to find the best partner to use quinolones in combination in malaria treatment (ACT for example).

## Tigecycline

Tigecycline is the lead of a new class of antimicrobials, the glycylcyclines, which belong to the tetracycline class. It is a semi-synthetic derivative of minocycline containing a glycyl amino substitution at position 9 [89]. Tigecycline is highly effective against gram-positive, gram-negative, atypical, anaerobic, and other difficult-to-treat pathogens. This drug is specifically designed to overcome two common mechanisms of tetracycline resistance, namely resistance mediated by acquired efflux pumps and/or ribosomal protection [90]. This tetracycline analogue is not recommended in children and pregnant women. Tigecycline with a twice-daily dosing regimen is generally well tolerated, but because it must be administered intravenously, its use in malaria treatment should be reserved for patients with severe and complicated malaria. The anti-malarial activity of tigecycline was first tested *ex vivo* on isolates from Bangladesh using the HRP2 ELISA assay [90]. Tigecycline showed significant activity correlation only with doxycycline and not with azithromycin, dihydroartemisinin, chloroquine, quinine, or mefloquine. These results suggested that tigecycline may have a delayed action on malaria parasites, just such as doxycycline, and appeared to be one of the best antibiotics against *P. falciparum*, with an IC_50_ within the nanomolar range and a relatively steep dose-response curve.

The in vitro activity of this molecule was then studied in clinical isolates of *P. falciparum* from Gabon [91]. As in the previous study, the activity was compared with that of clindamycin and doxycycline. This study demonstrated the substantial in vitro activity of tigecycline on *P. falciparum*. Tigecycline appeared to act faster than any other tetracyclines, with the highest activity at day 3. Meanwhile, the study underlined the limited clinical use of tigecycline due to its pharmacokinetic properties [92], with a risk to expose parasite populations to a prolonged period of subtherapeutic concentrations, thus increasing the risk of resistance.

All these results were confirmed in South America with the evaluation of in vitro antimalarial activity of tigecycline against culture-adapted reference strains and clinical isolates from the Brazilian Amazon [93]. However, all these in vitro studies were performed on a limited number of isolates; in vivo assays and randomized clinical trials are needed to establish the clinical applicability of tigecycline. Moreover, the co-administration of tigecycline with a schizonticide drugs that have a short elimination half-life should be performed to observe a potential synergistic effect. A low dose of 3.7 mg/kg/day for four days produced a 77–92% suppression in parasitaemia on day 5 after the treatment of mice infected with *P. berghei*. The same report obtained a complete cure of malaria in *P. berghei*-infected mice with tigecycline (3.7 mg/kg/day) in combination with a subcurative dose of chloroquine (33.3 mg/kg/day) [94]. These results indicate the promising anti-malarial effect of glycylcyclines in combination with chloroquine and support further in vivo assays and randomized clinical trials.

Because tigecycline must be administered intravenously, its use in malaria treatment should be reserved for patients with severe and complicated malaria. With the emergence of resistance to artesunate and ACT in Southeast Asia [95, 96], tigecycline could be a partner to artesunate in complicated malaria.

## Mirincamycin

Mirincamycin is a lincosamide antibiotic similar to clindamycin that is synthetically produced. This older molecule was studied in 2009 on *P. falciparum* isolates from Gabon [97]. In this study, the inhibitory activities of cis- and trans-mirincamycin (3.2 and 2.6 nM, respectively) were compared with the activities of doxycycline (720 nM) and clindamycin (12 nM). The study reported high in vitro activity against clinical *P. falciparum* isolates. IC_50_ of both isomers were substantially lower than those of any other antibiotic tested so far, including the lincosamide comparator clindamycin [97]. Myrancamycin showed in vitro additive and synergistic effects in combination with tafenoquine, dihydroartemisinin and chloroquine [98]. Furthermore, in *Plasmodium cynomolgi* infections of rhesus monkeys, mirincamycin was curative as a monotherapeutic regimen and showed an addictive effect when given together with primaquine [99, 100]. However, the role of mirincamycin on pre-erythrocytic stages is unclear. It failed to kill hypnozoites in infected rhesus monkeys with *P. cynomolgi* [101]. At 80 mg/kg/day for seven days orally, mirincamycin did not prevent relapse in *P. cynomolgi*-infected monkeys while it inhibited pre-erythrocytic development of *P. cynomolgi* at 40 mg/kg in another study [99]. This might be due to its low oral bio-availability in monkeys (<15%) [102]. In these studies, toxicity was reported to be similar to that of clindamycin. After several years, further clinical developments of mirincamycin are being pursued, and the molecule seems to be an interesting partner to fast-acting anti-malarial drugs.

## Ketolide agents

Ketolides are a recent class of macrolide derivative agents characterized by the replacement of the 3-cladinose of the macrolide ring by a keto-group. Ketolides inhibit bacterial growth by interfering with the translation of messenger RNA [103, 104]. These antibiotics present a relatively large antimicrobial spectrum and significantly accumulate in tissues. An in vitro study performed in South Africa determined the anti-plasmodial activity of two ketolide agents, including telithromycin, against chloroquine-susceptible and chloroquine-resistant strains of *P. falciparum* (3–15 nM) [105]. Telithromycin induced a delayed effect in *P. falciparum* parasites, suggesting its implication in the impairment of the apicoplast translation processes [106]. Cethromycin, a macrolide-quinoline hybrid, at a dose of 12 mg/kg in combination with primaquine at a dose of 15 mg/kg showed greater than 99% *P. yoelii* elimination on infected mice [107]. Tricyclic ketolides with anti-malarial activity were inhibitors of histone deacetylase protein (HDAC) [108]. These results indicated that, anti-malarial potential of the ketolide antimicrobial agents should further be evaluated.

## Fusidic acid

Fusidic acid is a steroid antibacterial derived from the fungus *Fusidium coccineum* and used for methicillin-resistant *Staphylococcus aureus.* Its displayed an in vitro anti-malarial activity against *P. falciparum* parasites at concentrations achievable by oral administration [109]. Two studies showed the effect of the elongation factor-G (EF-G) inhibitor on the translation apparatus of two malaria parasite organelles (the mitochondrion and the apicoplast) [110, 111]. Fusidic acid stalls the EF-G/GDP complex by binding to this complex immediately after GTP hydrolysis and by inhibiting a conformational change required for the release of the factor from the ribosome. The investigation on recombinant apicoplast and mitochondrion of *P. falciparum* showed that inhibition of *P. falciparum* growth in erythrocytes by fusidic acid does not exhibit the classic ‘delayed death’ phenotype observed for apicoplast-targeted proteins. Indeed, the inhibition of the parasite occurred both in the first as well as the second cycle of infection of *P. falciparum* parasites in blood culture. Secondly, fusidic acid presented a greater inhibitory effect on the apicoplastic EF-G than on the mitochondrial EF-G. However, no clinical trial has been performed to evaluate the efficacy of fusidic acid on *P. falciparum* infections. Therefore, research for the precise target of such antibiotics is useful when designing future anti-malarial molecules and derivatives.

## Thiopeptides: thiostrepton and nocathiacin

Thiostrepton, a thiazolyl peptide or thiopeptide, is produced by *Streptomyces azureus*. Micrococcin, another thiopeptide, is produced by *Bacillus* and *Micrococus* spp. While thiostrepton displayed *P. falciparum* growth inhibition at a micromolar range, micrococcin acted at a nanomolar range [112, 113]. Thiostrepton exhibited gametocytocidal activity and interfered both with apicoplast and parasite proteasome [114–116]. Thiostrepton binds to the *P. falciparum* apicoplast 23S rRNA [117, 118]. However, thiostrepton showed effects on mitochondrial protein synthesis [119]. Thiostrepton exerted action on mitochondrial EF-G [110]. This drug could be a candidate against malaria in the future; thus, its mechanism of action needs to be better documented.

Nocathiacin, another thiazolyl peptide, displayed potent activity against a wide spectrum of multidrug resistant gram-positive bacteria and inhibited protein synthesis. A water-soluble derivative of nocathiacin exerted an irreversible growth inhibition within the first growth cycle at a nanomolar range against chloroquine-susceptible and resistant *P. falciparum* strains and was immediately effective [120]. Further investigations need to be done on this drug and its derivatives to understand its mode of action *P. falciparum*.

## Conclusions

If tetracyclines and macrolides are now well known as anti-malarial drugs, other antibiotics should also be considered. Major discoveries could arise from chemical modifications of older molecules with anti-plasmodial properties.

An advantage of using antibiotics already approved, like doxycycline, tigecycline, clindamycin, azithromycin or co-trimoxazole, as anti-malarial drugs is the reduced cost of clinical development. Additionally, most of the antibiotics already approved are inexpensive and almost universally available. Another advantage is that the modes of action of antibiotics (action on apicoplast, inhibition of type II topo-isomerase enzyme, *P. falciparum* bc_1_ complex, PfNDH2, DHODH or HDAC) differ from those of most currently used drugs. This difference in modes of action implies that there are not cross-resistance between antibiotics and standard anti-malarial drugs. Antibiotics can be used in areas where parasites are resistant to standard anti-malarial drugs. This difference in modes of action also implies that antibiotics can be a good partner for combination. Clindamycin is recommended by the WHO in combination with quinine for the treatment of uncomplicated malaria in pregnant women during the first trimester [121]. Additionally, some of the new antibiotics show synergistic effects in combination with standard anti-malarial drugs: myrancamycin showed in vitro synergistic effects in combination with tafenoquine, dihydroartemisinin and chloroquine [98] and ELQ-300 was highly synergistic with atovaquone in *P. yoelii* murine models [122]. Nevertheless, the greater use of antibiotics against malaria cannot be promoted without considering the risk of the emergence of resistant bacteria. The use of antibiotics for malaria chemoprophylaxis always triggers opposition from a number of bacteriologists, who note the risk of selecting resistant bacteria cyclins [123]. The impact of chemoprophylaxis by doxycycline on bacterial pathogens is the best documented. In 1988, a publication reported tetracycline-resistant cases of *Campylobacter jejuni* gastroenteritis among American soldiers based in Thailand [124]. A subsequent study by the same team showed that taking doxycycline for malaria prophylaxis resulted in less exposure to resistant bacteria than the acquisition of already resistant s, which has long been widespread in this country [125]. The increase in multidrug-resistant gram-negative bacteria colonization among US military personnel in Afghanistan is likely due to environmental exposures rather than doxycycline exposure [126]. Methicillin-susceptible *Staphylococcus aureus* and methicillin-resistant *Staphylococcus aureus* colonization of military personnel under deployment was not associated with doxycycline exposure [127]. However, outbreaks of Panton-Valentine leukocidin-positive, doxycycline resistant, methicillin-susceptible *Staphylococcus aureus* infections associated with doxycycline prophylaxis have been reported in the French Army at the Ivory Coast [128]. Except for these military clinical cases, no study has been published about the risk of bacterial resistance to tetracyclines associated with their prophylaxis use. In the light of these observations, the impact of malaria chemoprophylaxis with doxycycline on bacteria resistance is not clear. Additionally, microbiome plays an important role in human health. Changes in the microbial flora can promote resistance or infection by pathogenic bacteria. Antibiotics have an impact on the microbiota that can lead to the spread of the pathogen populations [129, 130]. Q fever endocarditis patients treated with doxycycline presented significantly lower concentrations of *Bacteroidetes*, *Firmicutes* and *Lactobacillus* [131]. However, the same observation was found with hydroxychloroquine [131]. However, development of resistance in bacterial pathogens cannot be excluded in wide use of antibiotics for the treatment of uncomplicated malaria. The use in malaria treatment of antibiotics should perhaps be reserved for patients with severe and complicated malaria or patients from special risk groups with uncomplicated malaria. The use of antibiotics should only be considered after reviewing the conclusive results of clinical trials performed on exposed populations from different geographical areas.

## Abbreviations

ACT: artemisinin-based combination therapy
AIDS: acquired immune deficiency syndrome
DHODH: dihydroorotate dehydrogenase
DNA: deoxyribonucleic acid
EF-G: elongation factor-G
GTP: guanosine triphosphate
HDAC: histone deacetylase protein
HIV: human immunodeficiency virus
HRP2: histidine-rich protein II
IC_50_: inhibitory concentration 50%
IPT: intermittent preventive treatment
PfNDH2: *P. falciparum* type II NADH: ubiquinone oxydoreductase
PfSSB protein: *P. falciparum* single-stranded DNA binding protein
RNA: ribonucleic acid
WHO: World Health Organization
